# Effects of Hypoxia on the Antibacterial Activity of Epidermal Mucus from Chilean Meagre (*Cilus gilberti*)

**DOI:** 10.3390/ani14132014

**Published:** 2024-07-08

**Authors:** Belinda Vega, Teresa Toro-Araneda, Juan F. Alvarado, Claudia B. Cárcamo, Fanny Guzmán, Félix Acosta, Marcia Oliva, Edison Serrano, Janeth I. Galarza, Claudio A. Álvarez

**Affiliations:** 1 Laboratorio de Fisiología y Genética Marina (FIGEMA), Centro de Estudios Avanzados en Zonas Áridas (CEAZA), Coquimbo 1781421, Chile; teresa.toro@alumnos.ucn.cl (T.T.-A.); claudia.carcamo@ceaza.cl (C.B.C.); 2 Facultad de Ciencias Agronómicas, Universidad de El Salvador, San Salvador 3110, El Salvador; francisco.panameno@ues.edu.sv; 3 Marine Department, Tilad Group, Riyadh 12821, Saudi Arabia; 4 Núcleo Biotecnología Curauma (NBC), Pontificia Universidad Católica de Valparaíso, Valparaíso 2373223, Chile; fanny.guzman@pucv.cl; 5 Grupo de Investigación en Acuicultura (GIA), Instituto Universitario Ecoaqua, Universidad de Las Palmas de Gran Canaria, 35214 Taliarte, Spain; felix.acosta@ulpgc.es; 6 Laboratorio de Cultivo de Peces Marinos, Departamento de Acuicultura, Facultad de Ciencias del Mar, Universidad Católica del Norte, Coquimbo 1781421, Chile; moliva@ucn.cl (M.O.);; 7 Centro de Investigaciones Biológicas y Prácticas Académicas, Facultad de Ciencias del Mar, Universidad Estatal Península de Santa Elena, La Libertad 240204, Ecuador

**Keywords:** aquaculture, *Cilus gilberti*, hypoxia, mucosal immunity, skin mucus, antibacterial activity, *Vibrio* spp.

## Abstract

**Simple Summary:**

The Chilean meagre (*Cilus gilberti*) has been domesticated to enhance aquaculture in South America. However, hypoxia, a known chronic stressor in fish that affects their immune response to pathogens, could pose a significant challenge. This study focused on evaluating the impact of acute and intermittent hypoxia on the antibacterial capacity of the epidermal mucus of *C. gilberti*. Overall, the mucus of fish under both conditions exhibited activity against two marine pathogens belonging to the genus *Vibrio* sp. During hypoxia events, this activity decreased, but upon restoration of normal oxygen concentration, mucus activity levels recovered to match those of the normoxia group. Notably, oxygen deficiency affected the innate immune response of fish to the lipopolysaccharide (LPS) of *Vibrio anguillarum*. While LPSs stimulated mucus activity under normoxic conditions, no stimulation and even downregulation were observed in fish exposed to hypoxic conditions. Lysozyme displayed a similar pattern of activity, while no modulation of peroxidase activity was detected after periods of hypoxia. This work constitutes the first study about the environmental impact on the defense mechanisms of *C. gilberti*. It aims to contribute to enhancing the welfare and health of cultured fish, thereby fostering greater productivity within the future Chilean meagre aquaculture industry.

**Abstract:**

Comprehending the immune defense mechanisms of new aquaculture species, such as the Chilean meagre (*Cilus gilberti*), is essential for sustaining large-scale production. Two bioassays were conducted to assess the impact of acute and intermittent hypoxia on the antibacterial activity of juvenile Chilean meagre epidermal mucus against the potential pathogens *Vibrio anguillarum* and *Vibrio ordalii*. Lysozyme and peroxidase activities were also measured. In general, fish exposed to hypoxia showed a 9–30% reduction in mucus antibacterial activity at the end of hypoxic periods and after stimulation with lipopolysaccharide. However, following water reoxygenation, the activity of non-stimulated fish was comparable to that of fish in normoxic conditions, inhibiting bacterial growth by 35–52%. In the case of fish exposed to chronic hypoxia, the response against *V. anguillarum* increased by an additional 19.8% after 6 days of control inoculation. Lysozyme exhibited a similar pattern, while no modulation of peroxidase activity was detected post-hypoxia. These results highlight the resilience of *C. gilberti* to dissolved oxygen fluctuations and contribute to understanding the potential of mucus in maintaining the health of cultured fish and the development of future control strategies.

## 1. Introduction

The growth experienced by the aquaculture sector in recent years has spurred the search for new species suitable to be cultivated. A notable example is Chile, where government initiatives like the Diversification of Chilean Aquaculture Program (PDCh) aim to domesticate native species such as the Chilean meagre (*Cilus gilberti*; Abbott 1899). This marine species, belonging to the *Sciaenidae* family, is native to the coastal waters of Peru and Chile (6° S–43° S), including the Galápagos Islands in Ecuador [1,2]. However, the captive rearing of marine organisms often leads to the emergence of diseases, which tend to intensify and diversify alongside the production processes [3,4].

Though potential bacterial pathogens of cultivated *C. gilberti* have not been described yet, international aquaculture experiences with sciaenids indicate their susceptibility to Gram-negative bacteria, such as *Vibrio* spp. [5,6], causing significant economic losses worldwide due to high mortality rates [7,8]. Despite fish having a complete immune system capable of generating innate and adaptive responses, they heavily rely on their innate defense system for protection against infections [9,10]. Therefore, early detection of these microorganisms is vital for fish survival.

Fish mucosal organs, including skin, gills, intestines, and nasal mucosa, continuously interact with the aquatic microbiota and the external environment, serving as the primary route of pathogen entry [11,12]. Consequently, these tissues constitute the animals’ first line of defense [13,14], containing epithelial and mucus-producing cells that form a physicochemical barrier hindering pathogenic microorganism adhesion. They also facilitate the action of innate and adaptive immune components, such as lectins, transferrins, immunoglobulins (IgM), complementing antimicrobial proteins and enzymes [15,16,17]. Moreover, they harbor mucosa-associated lymphoid tissue (MALT) housing cells that express an array of pathogen recognition receptors (PRRs) capable of identifying the pathogen-associated molecular patterns (PAMPs) present in microorganisms [18], inducing the host’s immune response. Some common PAMPs in Gram-negative bacteria include lipopolysaccharides (LPS), peptidoglycans (PGN), flagellin, porin, and proteins from secretion systems, like type III (T3SS) [19,20]. Among the leukocytes within MALT, macrophages play a pivotal role in killing pathogens through phagocytosis, effectively destroying microorganisms within cells [16,21,22,23]. This process is facilitated by the production and action of innate effectors such as reactive oxygen species (ROS) and AMPs. Particularly, the mucosa of teleost fish has been found to contain the AMPs cathelicidins, hepcidins, piscidins, and β-defensins [24].

The strategic plan for *C. gilberti* farming in Chile involves relocating juveniles to northern regions with higher water temperatures. However, records from experimental cage rafts in these areas revealed frequent dissolved oxygen (DO) concentration variations in the sea, sometimes dropping below 2.5 mg L^−1^ [25,26,27]. Reasons for these changes may include El Niño oceanographic oscillations, proximity to oxygen minimum zones (OMZs; <0.64 mg/L), and upwelling of water masses with low levels of DO [28,29,30]. These phenomena are exacerbated by the climate changes observed since the mid-19th century, impacting water quality parameters in aquaculture areas and presenting new challenges for both existing and new species aquafarming. In this sense, temperature changes, pH, salinity fluctuations, and variations in oxygen availability are becoming more frequent, favoring the proliferation of pathogenic microorganisms [31]. Mucosal immune responses to pathogens can also be significantly altered by physicochemical changes in water [9,32]. Particularly, oxygen deficiency negatively affects the innate immune system of teleost fish, increasing oxidative stress and affecting both local and systemic defense responses, ultimately rendering the fish vulnerable to opportunistic pathogens [33]. Therefore, understanding the effects of these changes on mucosal immune responses is crucial in ensuring proper fish development in fish farming cages.

Therefore, the objective of this study was to ascertain the defensive function of *C. gilberti* epidermal mucus against potential pathogens and assess the effects of hypoxia stress. Its proper understanding will play a crucial role in preserving fish health under farming conditions and developing future prophylactic control strategies, such as the proper use of vaccines or immunostimulants for new marine species of aquaculture.

## 2. Materials and Methods

### 2.1. Animals

Fish were supplied by Fundación Chile (FCh) and transferred to the facilities of the Faculty of Marine Sciences at the Universidad Católica del Norte (UCN), where subsequent in vivo experiments were conducted. The fish were maintained in 250 L tanks at a density of 20 kg/m^3^ in seawater recirculating aquaculture systems (RAS) at 14.5 °C, pH 7.7, and 7.8 mg/L DO concentration. The photoperiod was approximately 11 h of light and 13 h of darkness, and fish were fed twice a day with a commercial diet at 1% of biomass. Both the animals and the physicochemical parameters of the water were monitored daily. All the experiments on fish were approved by the Ethics Committee of Universidad Católica del Norte Sede Coquimbo.

### 2.2. Acute Hypoxia and Bath Challenge

**Figure 1:**
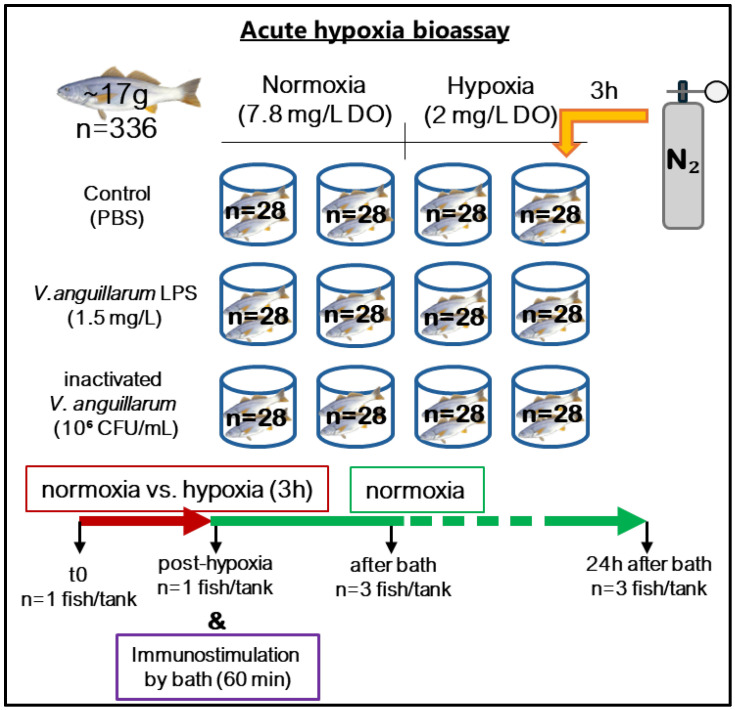
Experimental design for the acute hypoxia bioassay.

For the acute hypoxia bioassay (Figure 1), a total of 336 corvina juveniles (~17 g) were randomly distributed into 12 80 L conical tanks in groups of 28 fish in seawater RAS in similar conditions to those described previously. After a two-week acclimatization period, the fish were initially divided into two experimental groups, each consisting of 6 tanks. The fish from the control group were kept under normoxic conditions (7.8 mg/L DO), while those in the second group were exposed to 3 continuous hours of hypoxia. The air and water flows were interrupted in the second group, and then nitrogen (N_2_) was injected into the water until a DO concentration of 2 mg/L was reached. Subsequently, normal air and water flows were restored, and DO levels returned to the normoxic state.

For bath challenges, the water volume in the tanks was reduced to 30 L. Following this, both normoxic and hypoxic groups were further divided into three subgroups that were (i) inoculated with LPS from *V. anguillarum* (1.5 mg L^−1^), (ii) formalin-inactivated *V. anguillarum* (10^6^ CFU mL^−1^), and (iii) inoculated with 10 mM phosphate buffer saline (PBS).

Epidermal mucus from 6 fish per experimental group was collected at the beginning of the experiment, after 3 h of exposure to hypoxia and after 0 and 24 h of immunostimulation, respectively. Before sample collection, the fish were anesthetized with AQUI-S^®^-isoeugenol 50% (Bayer, Leverkusen, Germany). Additionally, the weight and size of the animals were recorded.

### 2.3. Chronic Hypoxia and LPS Challenge

**Figure 2:**
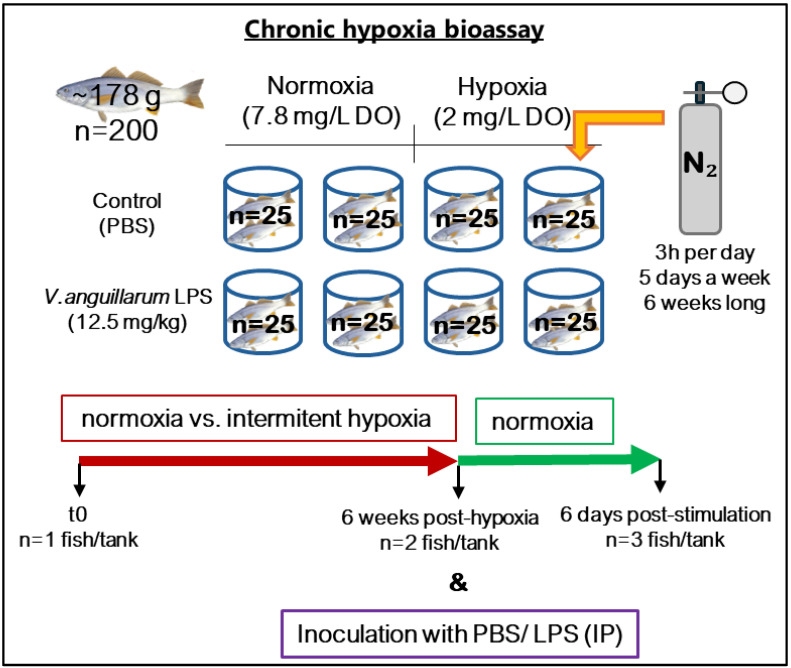
Experimental design of the chronic hypoxia bioassay.

A second in vivo experiment was conducted to assess the impact of chronic hypoxia (Figure 2). In this instance, 200 corvina juveniles (~178 g) were randomly distributed into eight 300 L tanks, divided into two groups of four tanks each, with 25 fish per tank. Throughout the experiment, the control group remained under normoxic conditions (7.8 mg/L DO), while the second group was subjected to intermittent hypoxia events (2 mg/L DO) for 3 h per day, 5 days a week, over a period of 6 weeks, aiming to simulate the environmental conditions at Chilean meagre cultivation sites. The reduction in DO concentration in the water was achieved using the same procedure described in the previous experiment. At the end of this period, the fish under both normoxic and hypoxic conditions were subdivided into two groups: stimulated and non-stimulated (control group). The stimulated group were intraperitoneally (IP) inoculated with LPS of *V. anguillarum* (12.5 mg kg^−1^ body mass) to induce their innate immune response, while the control group was IP injected with a same volume of PBS.

Prior to sample collection, the fish were anesthetized (AQUI-S^®^-isoeugenol 50%) and their weight and size were recorded. Subsequently, skin mucus samples were collected from 8 fish (one per tank) at the beginning of the trial, from 8 fish per group after 6 weeks under intermittent hypoxia, and from 6 fish per treatment after 6 days of immunostimulation.

### 2.4. Mucus Sample Recovery and Total Protein Extraction

The mucus was meticulously gathered from the fish skin by gently dragging along the dorsal area from cranial to caudal and then being carefully placed in a 50 mL tube. Each pool of samples (from six juvenile Chilean meagre), comprising mucus from all animals within the same group, was kept on ice during collection and subsequently preserved at −80 °C until further use. Following this, the mucus samples were thawed on ice and subjected to centrifugation to precipitate and remove scales and other undesirable components. Subsequently, the proteins from the mucus were extracted.

The mucus was homogenized in a lysis buffer (50 mM Tris-HCl, 10 mM EDTA, pH 8) containing a commercial protease inhibitor (Sigma-Aldrich, St. Louis, MO, USA), utilizing a Mini-Beadbeater-24 Biospec apparatus. Subsequently, the homogenized mixture underwent centrifugation at 12,000× *g* for 15 min at 4 °C. The supernatant containing the proteins was collected, lyophilized, and stored at −20 °C.

The quantification of the total proteins extracted was performed using the colorimetric bicinchoninic acid (BCA) method following the commercial protocol (Pierce™ BCA kit, Waltham, MA, USA). Then, the protein extracts were reconstituted in Milli-Q water at a stock concentration of 2 mg mL^−1^.

### 2.5. Antibacterial Activity

Two pathogenic strains of *Vibrio* spp., namely *V. anguillarum* 507 and *V. ordalii* DSM 19621, were selected to explore the antibacterial potential of corvina mucus against them. Both strains were cultivated on tryptic soy agar (BD) supplemented with 1.5% sodium chloride (TSA-1.5% NaCl) at 25 °C for 24 h. Following this, a single colony was inoculated into 20 mL of tryptic soy broth (BD) (TSB-1.5% NaCl) and incubated for 16 h at 25 °C under constant agitation (80 rpm). The optical density of the cultures was measured at a wavelength of 600 nm (Table 1), and both bacterial strains were adjusted to a concentration of 10^7^ CFU mL^−1^.

The antibacterial activity of the mucus was determined through a cell viability assay, specifically, by the reduction of MTT (3-(4,5-dimethylthiazol-2-yl)-2,5-diphenyltetrazolium bromide) tetrazolium [34]. This method is based on the capability of viable cells to convert MTT into formazan, an insoluble purple-colored compound. Conversely, non-viable cells lose this capacity, resulting in a yellow color indicative of the absence of live cells with active metabolism. Accordingly, 100 µL of mucus total protein extract was added to 100 µL of bacterial culture (1.00 × 10^6^ CFU) in 2 mL tubes at a final concentration of 0.5 mg mL^−1^. The growth control consisted of a bacterial culture without mucus extract, while for the activity control, gentamicin (Gibco, Billings, MT, USA) was applied to the cultures at 0.5 mM. Finally, a 1:1 mixture of water and TSB-1.5NaCl served as a blank. Each sample was prepared in triplicate and maintained at 25 °C for 5 h under low agitation (60 rpm). Following this incubation period, 25 µL of MTT (1 mg mL^−1^; Sigma-Aldrich) was added to each tube, followed by an additional 10 min incubation at 25 °C. Subsequently, 200 µL of dimethyl sulfoxide (DMSO) was employed to dissolve the formazan, and 200 µL of each sample, in duplicate, was dispensed into a flat-bottom 96-well plate. The amount of formazan, directly proportional to the number of viable bacteria, was quantified by measuring absorbance at 570 nm using a microplate reader (Epoch™, Santa Monica, CA, USA). Finally, bacterial survival was calculated as the difference between the absorbance of the sample and that of the blank [35]. In addition, the antibacterial activity of mucus extracts was expressed as a percentage of inhibition (%), determined from the bacterial survival in each sample relative to the bacterial growth control (100% survival or 0% inhibition) [34].

### 2.6. Lysozyme Activity

The turbidimetric determination of lysozyme activity in the skin mucus of fish involved measuring the reduction in absorbance over time due to the lysis of *Micrococcus lysodeikticus* [36,37]. Briefly, 50 µL of each sample, comprising 100 µg of protein extracts, along with 50 µL of Milli-Q water in the blanks, were added in duplicate to a flat-bottom 96-well plate. Concurrently, commercial lysozyme from chicken egg white (USBiological, Salem, MA, USA) was prepared at a concentration of 2 mg mL^−1^ in Milli-Q water, and serial 1:2 dilutions were performed to construct the standard curve. Furthermore, lyophilized *Micrococcus* sp. (Sigma, Kawasaki, Japan) was resuspended in PBS 1× pH = 7.0 at a concentration of 0.3 mg mL^−1^ to be used as the enzyme substrate. Immediately thereafter, 200 µL of this suspension was added to each well, and optical density was measured in a microplate reader (Epoch, Biotek, Winooski, VT, USA) at a wavelength of 450 nm every minute for 5 min.

One lysozyme unit of activity was defined as the amount of enzyme that induces a reduction in absorbance of 0.001 min^−1^. The enzymatic activity of the analyzed samples was expressed in lysozyme activity units per milliliter of mucus total protein extract (U mL^−1^) and calculated based on the slope of each line obtained from the set of readings at the specified times. The OD_450_ of the wells corresponding to blanks, i.e., *Micrococcus lysodeikticus* suspension without mucus extract, usually remained stable during measurements. If noticeable differences were observed, the slope value of controls was subtracted from that of the samples. Additionally, the amount of egg lysozyme equivalent to the enzymatic activity in the mucus samples was determined using the linear regression equation resulting from the calibration curve.

### 2.7. Peroxidase Activity

To assess the protective role of fish mucus against oxidative damage during the respiratory burst for pathogen elimination in macrophages, the enzymatic activity of glutathione peroxidase was determined using a colorimetric assay based on the reduction of hydrogen peroxide (H_2_O_2_). For this, 50 µL of each total protein extract from the mucus was added in duplicate to a flat-bottom 96-well microplate, with distilled water as the blank. Subsequently, 50 µL of chromogenic reagent 3,5,3′5′-tetramethylbenzidine (TMB) (20 mM) and 50 µL of H_2_O_2_ (5 mM) were added as enzyme substrates. TMB acts as a hydrogen donor for the reduction of H_2_O_2_ to water (H_2_O), resulting in a blue color solution. After 2 min, the reaction was halted by adding 50 µL of H_2_SO_2_ (2 M), turning TMB yellow. Finally, absorbance was measured at 450 nm using a plate reader (Epoch) [15,38].

One unit of peroxidase activity was defined as the amount of enzyme causing an absorbance change of 1, and the activity was expressed as U mg^−1^ mucus proteins. Furthermore, the amount of peroxidase present in the samples was calculated based on a calibration curve prepared with commercial peroxidase (Sigma-Aldrich). The curve was generated using serial dilutions (1:2) from a starting concentration of 0.1 mg mL^−1^.

### 2.8. Statistical Analysis

The results are expressed as the mean ± standard deviation (mean ± SD). Statistical differences between groups were assessed through one-way analysis of variance (ANOVA), followed by Bonferroni’s post hoc test for the identification of specific differences. The normality of the data was preliminarily evaluated using the Shapiro–Wilks test. All statistical analyses were conducted using IBM SPSS Statistics 29.0 and GraphPad Prism 10. Significance was reported at *p* ≤ 0.05.

## 3. Results

### 3.1. Antibacterial Activity

**Figure 3:**
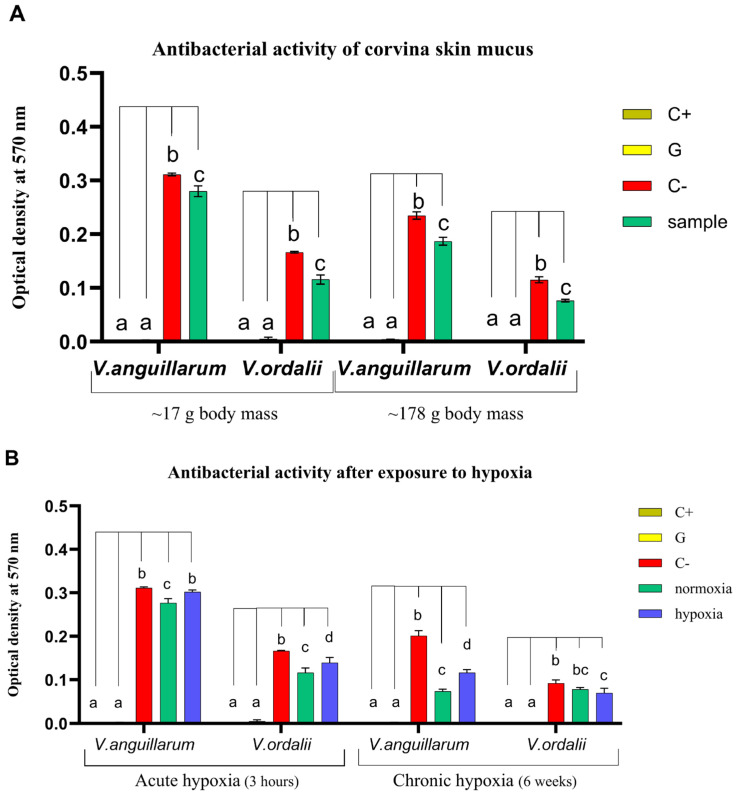
Survival rate of *V. anguillarum* and *V. ordalii* against total protein extracts from *C. gilberti* epidermal mucus of juveniles weighing ~17 and ~178 g body mass (**A**) under normoxic and (**B**) hypoxic (acute and chronic) conditions. Data are expressed as optical density at 570 nm and presented as mean ± SD (n = 3). Index: C+: blank; G: gentamicin 50 µM (activity control); C−: bacterial growth control; normoxia: mucus extracts from fish in the normoxia group (7.8 mg L^−1^ DO); hypoxia: samples from fish under the acute or intermittent hypoxia condition (2 mg L^−1^ DO). Different letters indicate statistically significant differences between groups (*p* ≤ 0.05).

Cell viability assays demonstrated that the epidermal mucus of juvenile corvina exhibited significant antibacterial activity against *V. anguillarum* (*p* < 0.0005) and *V. ordalii* (*p* < 0.0001). A concentration of 0.5 mg mL^−1^ of total protein mucus extract effectively inhibited the growth of both bacterial strains, each at a concentration of 1.00 × 10^7^ CFU mL^−1^, after a 5 h exposure period (Figure 3A). In particular, the mucus collected at the outset of the experiments displayed an inhibition of approximately 30% in the growth of *V. ordalii* for both 7- and 15-month-old juveniles. Notably, when confronted with *V. anguillarum*, the inhibition doubled (20.5%) in the older fish (Figure 3A). It is worth noting that these percentages exhibited variability throughout the experiment, as will be detailed later (Figure 3B). The bacterial growth inhibition was evidenced by the lower optical density recorded in the samples compared to their respective growth controls (bacterial cultures without mucus extract).

When assessing the impact of hypoxia on corvina response, a reduction of 28.3% and 14% was observed in antibacterial activity against *V. anguillarum* and *V. ordalii,* respectively, of fish exposed for 3 h to low oxygen levels, in comparison to those maintained under normoxic conditions (*p* < 0.04). Specifically, the mucus from the hypoxia-exposed group exhibited antibacterial activity, with a 16.2% growth inhibition against *V. ordalii* (*p* < 0.02), while completely losing it against *V. anguillarum*. In contrast, the normoxia group showed consistent mucus activity of 30–31% against both bacterial strains (Figure 3B).

After 6 weeks of intermittent hypoxia exposures, a significant inhibition of *V. anguillarum* bacterial growth was observed in both normoxia (63.5%) and hypoxia (42.3%) experimental groups (*p* < 0.0001). Notably, there was a 21.2% decrease in mucus activity in the hypoxic condition (*p* = 0.0001). Interestingly, chronic stress, unlike acute stress, did not cause a reduction of mucus activity against *V. ordalii* in the hypoxia-exposed group (24.8%; *p* < 0.02). The bacterial growth inhibition of hypoxia and normoxia fish (15.3%) showed no significant differences (Figure 3B).

**Figure 4:**
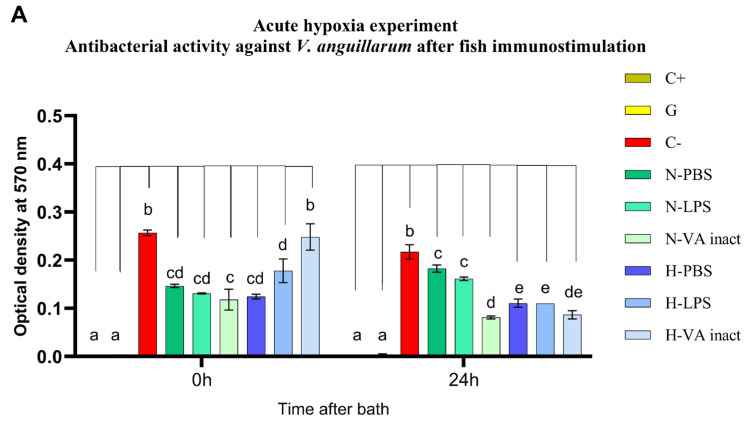

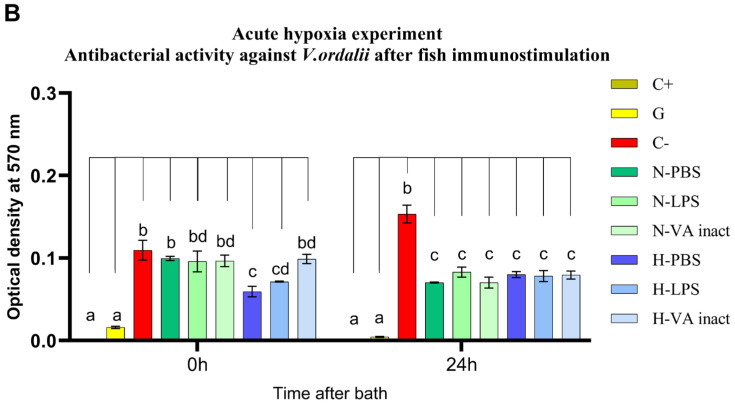
Survival rate of *V. anguillarum* (**A**) and *V. ordalii* (**B**) against total protein extracts from *C. gilberti* epidermal mucus after 0 and 24 h of fish immunostimulation by bath, previously exposed to normoxic and acute hypoxic conditions. Data are expressed as optical density at 570 nm and presented as mean ± SD (n = 3). Index: C+: blank; G: gentamicin (activity control); C−: bacterial growth control; N: normoxia group; H: hypoxia group; PBS: non-stimulated group; LPS: group stimulated with lipopolysaccharide of *V. anguillarum*; VA inact.: group stimulated with formalin-inactivated *V. anguillarum*. Different letters indicate statistically significant differences between groups (*p* ≤ 0.05).

**Figure 5:**
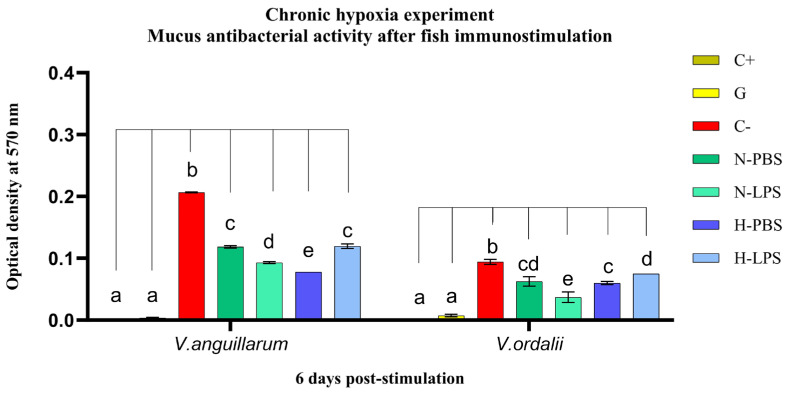
Survival rate of *V. anguillarum* and *V. ordalii* against total protein extracts from *C. gilberti* epidermal mucus after 6 days of fish immunostimulation, previously exposed to normoxic and chronic hypoxic conditions. Data are expressed as optical density at 570 nm and presented as mean ± SD (n = 3). Index: C+: blank; G: gentamicin (activity control); C−: bacterial growth control; N: normoxia group; H: hypoxia group; PBS: non-stimulated group; LPS: fish intraperitoneally inoculated with LPS of *V. anguillarum*. Different letters indicate statistically significant differences (*p* ≤ 0.05).

Subsequently, fish were inoculated with LPS from live *V. anguillarum* or inactivated *V. anguillarum*, to stimulate the immune response of Chilean meagre. Interestingly, LPS administered by bath in the normoxic condition group did not change the antibacterial activity of the skin mucus (Figure 4). In contrast, intraperitoneal inoculation of LPS resulted in a notable increase of 12.4% and 27.2% in mucus activity against *V. anguillarum* (*p* < 0.0001) and *V. ordalii* (*p* < 0.002), respectively. This can be compared with the activity exhibited by the non-stimulated fish against both strains: 42.7% inhibition of *V. anguillarum* and 33.3% inhibition of *V. ordalii* (*p* < 0.0001) (Figure 5). Additionally, a 62.8% reduction in the bacterial concentration of *V. anguillarum* was observed after 24 h of bath stimulation with inactivated bacteria (*p* < 0.0001) (Figure 4A), while both stimulated and non-stimulated fish exhibited an average activity of approximately 50% against *V. anguillarum* at 0 h post-bath and against *V. ordalii* at 24 h post-bath (Figure 4).

Furthermore, the antibacterial capacity of mucus from fish exposed to both acute and chronic hypoxia experienced a ‘rebound effect’ after water and fish reoxygenation (Figure 4 and Figure 5), while mucus collected during low oxygen events generally exhibited lower inhibition of bacterial growth compared to that of fish kept under normoxia (Figure 3B). After the hypoxia periods, non-stimulated fish produced mucus with equal or higher antibacterial effect than that of the normoxia group after just 1 h of returning to normoxic conditions (duration of PBS bath) (Figure 4). This behavior was observed at all times studied, regardless of the duration of the previous hypoxia periods (Figure 4 and Figure 5).

In this context, it is relevant to highlight that the analysis of the protein extract from the mucus of fish exposed to 3 h of hypoxia and inoculated with PBS revealed 45.8% inhibition of *V. ordalii* growth at 0 h post-bath and 49.1% inhibition of *V. anguillarum* growth at 24 h post-bath. Consequently, this implies an increase of 36.8% and 33.1% in mucus activity compared to the normoxia group (*p* < 0.002 and *p* < 0.0001), respectively. Comparable inhibition percentages were observed for both *V. anguillarum* at 0 h post-bath (51.6%) and *V. ordalii* at 24 h post-bath (48.1%), aligning with those found under the opposing condition (Figure 4).

Similarly, the mucus from non-stimulated fish exposed to chronic hypoxia exhibited a 19.8% higher activity than that shown by the normoxia group (42.7%) against *V. anguillarum* (*p* < 0.0001). Conversely, the response against *V. ordalii*, resulting in a 36.3% inhibition of bacterial growth, was not significantly different than that in the normoxia condition (Figure 5). These results indicate a reduced effect of both acute and chronic hypoxia on the antimicrobial mucus response to *V. ordalii*.

However, fish that had experienced one hypoxic event and were subsequently inoculated with the inactivated pathogen did not exhibit an immediate antibacterial response against both strains due to the total loss of mucus activity. This loss of activity resulted in an increase in bacterial growth of 49% for *V. anguillarum* (*p* = 0.0002) and 36.2% for *V. ordalii* (*p* < 0.003) compared to the non-stimulated group. Fish exposed to acute hypoxia, when inoculated with LPS, also experienced a decline in mucus antibacterial activity of 20.8% against *V. anguillarum* (*p* > 0.2) and 10.8% against *V. ordalii* (*p* > 0.99). Nevertheless, they preserved an activity level of 30.7% and 35.1% against *V. anguillarum* and *V. ordalii* (*p* < 0.02 and *p* < 0.003). After 24 h of infection, the mucus of the fish recovered its normal activity, but in no case was it induced by *V. anguillarum* PAMPs (Figure 4).

A different scenario emerged in the chronic hypoxia experiment. While the stimulation of fish maintained under normoxic conditions induced an enhanced antibacterial response of the mucus (*p* < 0.0001), in fish exposed to low DO levels for 6 weeks and subsequently immunostimulated with LPS, the mucus activity diminished by 20.2% against *V. anguillarum* (*p* < 0.0001) and 15.8% against *V. ordalii* (*p* < 0.05). However, despite the observed reduction in activity, the mucus of the fish still exhibited significant antibacterial activity, inhibiting the growth of both *V. anguillarum* (*p* < 0.0001) and *V. ordalii* (*p* < 0.008) strains by 42.2% and 20.5%, respectively (Figure 5).

### 3.2. Lysozyme Activity

The lysozyme activity values, obtained by measuring 50 µL samples containing 100 µg of mucus total protein extract, are summarized in Table 2. In nearly all mucus samples collected from 7-month-old juveniles at the first two sampling points of the acute hypoxia experiment, no activity was detected. Enzymatic activity was only found in two samples at the low levels of 4.00 ± 5.66 U mL^−1^ in extracts from fish after exposure to hypoxia and 10.00 ± 2.83 U mL^−1^ after PBS bath in the fish maintained under normoxic conditions. Moreover, there were no statistically significant differences between the normoxia and hypoxia groups after 3 h of hypoxia and at 0 h after bath. Nevertheless, reoxygenation revealed a remarkable recovery of the antimicrobial response in fish previously exposed to an event of low oxygen concentration. This effect became particularly apparent when lysozyme activity, initially undetected after bath, reached 32.00 ± 0.00 U mL^−1^ in mucus extracts from non-stimulated fish at 24 h post-bath, also in contrast with an enzymatic activity of 4.00 ± 0.00 U mL^−1^ measured in the normoxia control group (*p* < 0.001). Interestingly, lysozyme synthesis remained unstimulated by both LPS and inactivated vaccine inoculation. Furthermore, in fish of the hypoxia group, LPS of *V. anguillarum* negatively modulated lysozyme activity after 24 h of administration (*p* = 0.0002). These results are similar to those observed previously in antibacterial activity assays (Figure 4).

On the other hand, exposing fish to intermittent hypoxia events over a 6 week period resulted in a reduction in lysozyme activity (*p* > 0.05), from 60.00 ± 11.31 U mL^−1^ to 30.00 ± 2.83 U mL^−1^. However, six days post the hypoxia period, the activity rebounded, showing higher levels in the non-stimulated animals from the hypoxia group (102.00 ± 36.77 U mL^−1^) compared to the normoxia group (54.00 ± 2.83 U mL^−1^) (*p* > 0.4). Although the differences were not statistically significant, this interestingly suggests once again that the return to normoxic conditions plays a pivotal role in the swift recovery of the antimicrobial potential of the Chilean meagre mucus. Furthermore, LPS of *V. anguillarum* induced an increase in lysozyme activity to 146.00 ± 8.49 U mL^−1^ exclusively in fish that were never subjected to low oxygen levels (*p* = 0.054). Conversely, a decreasing activity from 102.00 ± 36.77 U mL^−1^ to 70.00 ± 8.49 U mL^−1^ was observed in fish previously exposed to chronic hypoxia. In none of the cases were significant differences found when compared to their respective control groups (Table 2). Importantly, these findings match the results obtained from mucus antibacterial activity against *V. anguillarum* (Figure 5). Notably, the mucus of the 15-month-old fish exhibited higher levels of lysozyme activity compared to the mucus of the 7-month-old fish (Table 2).

### 3.3. Peroxidase Activity

The peroxidase activity in mucus extracts was 0.73 ± 0.42 U mg^−1^ in 17 g juveniles, contrasting with 3.26 ± 0.00 U mg^−1^ found in 178 g juveniles. Interestingly, acute hypoxia showed no impact on the enzymatic activity of the fish. Moreover, even after six weeks of intermittent exposure to hypoxia events, there was no detectable difference in peroxidase activity in the mucus. Nevertheless, an intriguing increase in activity, from 0.05 ± 0.01 U mg^−1^ to 0.65 ± 0.04 U mg^−1^, was detected in non-stimulated fish of the chronic hypoxia group, after six days without hypoxia events (<0.001).

Surprisingly, the peroxidase activity pattern in immunostimulated fish diverged from previous observations with lysozyme and antibacterial activities. Specifically, the inoculation with inactivated bacteria initially increased peroxidase activity from 0.20 ± 0.06 U mg^−1^ to 0.46 ± 0.01 U mg^−1^ in fish kept in normoxia (*p* < 0.01), and subsequently decreased after 24 h to 0.29 ± 0.06 U mg^−1^. Similarly, in fish exposed to acute hypoxia, enzyme activity rose from 0.33 ± 0.01 U mg^−1^ to 0.60 ± 0.01 U mg^−1^ (*p* < 0.01) after bath stimulation. This was followed by a more pronounced decrease after 24 h in the hypoxia condition, with enzyme activity levels dropping below those of non-stimulated fish (0.10 ± 0.01 U mg^−1^; *p* < 0.01).

Moreover, acute hypoxia inhibited the response of fish inoculated with LPS (*p* < 0.02), resulting in a decrease in peroxidase activity from 0.34 ± 0.02 U mg^−1^ to 0.14 ± 0.01 U mg^−1^ at 24 h post-administration. Notably, LPS also failed to induce a response in fish exposed to chronic hypoxia (*p* > 0.4). Meanwhile, LPS significantly stimulated peroxidase activity in the mucus of fish not previously exposed to low DO concentrations (*p* < 0.0001) in both experiments. Specifically, the bath administration of LPS to 7-month-old fish increased the enzyme activity from 0.30 ± 0.05 U mg^−1^ to 0.85 ± 0.03 U mg^−1^. Likewise, the intraperitoneal injection in 15-month-old fish resulted in an increase from 0.05 ± 0.01 U mg^−1^ to 2.57 ± 0.01 U mg^−1^. These results are presented in Table 3.

## 4. Discussion

Among the various defense mechanisms of fish against infections by pathogens, epidermal mucus plays a decisive role in maintaining their health condition. The integrity of the skin and the production of mucous secretions are crucial in establishing physical and chemical barriers that protect them from the external environment, primarily by trapping microorganisms, preventing their adherence to the epithelium, and subsequently stopping the invasion. Concurrently, it facilitates the action of innate immune components present in the mucus [11,15,16,39,40,41].

In this work, the mucus of *Cilus gilberti* exhibited antibacterial properties against *V. anguillarum* and *V. ordalii*, two pathogenic species of significant concern in marine aquaculture. Vibriosis, caused by these pathogens, also affects other sciaenids worldwide, such as *Argyrosomus regius* and *Larimichthys crocea* [5,6,7,8]. Furthermore, both bacterial strains have been identified as responsible for high morbidity and mortality in cultured salmonids in Chile [42,43]. Although disease susceptibility and immune responses vary considerably among different species [44], the lack of understanding of the immune system of new species, especially those introduced to aquaculture like *C. gilberti*, presents a limitation in developing immunological control strategies against infectious diseases in intensive culture systems. This challenge is further compounded when there are no prior studies addressing differences in their defense responses at different growth stages.

In the present study, we found that, irrespective of the age of the fish, the activity against *V. ordalii* remained unaltered, with the epidermal mucus reducing bacterial growth by 30%. However, an age-dependent activity was observed against *V. anguillarum*, as the mucus of younger fish exhibited lower antimicrobial activity against this pathogen. Likewise, lysozyme activity was also notably higher in 15-month-old fish, with enzymatic levels of 60.00 ± 11.31 U mL^−1^, while being undetectable or hardly detectable in 7-month-old juveniles. This extensively studied antimicrobial lytic enzyme has often been used as an indicator of the innate immune response in fish [45]. Previous research on cultured fish has revealed that lysozyme and AMPs are expressed in early developmental stages and play a pivotal role in fish survival against pathogens due to the immaturity of their immune systems [46,47,48]. Nevertheless, constitutive levels of lysozyme, AMPs, and immunoglobulin generally rise consistently with age and size [48,49,50]. These findings may offer an explanation for the above observations and might indicate a higher susceptibility of smaller juvenile fish to *V. anguillarum*. Other molecules that have germicidal and bactericidal functions are the ROS superoxide ions (O_2_-), and hydrogen peroxide (H_2_O_2_), produced endogenously by phagocytic cells during the respiratory burst for pathogen elimination. However, they also cause oxidative damage to the organism’s cells. Therefore, various antioxidant enzymes protect cells from this damage; among them, peroxidase acts on H_2_O_2,_ transforming it into harmless water and oxygen [51]. Again, the age, weight, and size of the fish influenced peroxidase activity in Chilean meagre, showing under normoxic conditions levels of 0.73 ± 0.42 U mg^1^ and 3.26 ± 0.00 U mg^−1^ in the mucus of 7- and 15-month-old juveniles, respectively.

Additionally, the intraperitoneal administration of *V. anguillarum* LPS for fish immunostimulation led to a notable enhancement in the antibacterial response of *C. gilberti* mucus. Lysozyme activity augmented from 54.00 ± 2.83 U mL^−1^ to 146.00 ± 8.49 U mL^−1^. Such increment, or a potential increase in mucous secretion alongside elevated levels of AMPs and immunoglobulins, as observed in teleost fish after infection and/or vaccination [46,48,52,53,54], could explain the 12.4% and 27.2% elevation over the mucus activity rate of non-stimulated *C. gilberti* against *V. anguillarum* and *V. ordalii*, respectively. These results may indicate modulation of the innate response by LPS. However, in Chilean meagre, LPS administration by bath did not induce the synthesis or activation of antibacterial components in mucus, despite facilitating direct contact between the polymer and mucous membranes, as naturally occurs. This suggests that the immersion method might not effectively deliver sufficient quantities of LPS to stimulate immunity [55]. Nonetheless, through this route, the inactivated bacteria triggered a 62.8% increase in mucus activity of meagre against *V. anguillarum* after 24 h. Previous studies have underscored the critical role of the administration route in vaccine effectiveness, with notably superior outcomes observed with injection [55,56,57]. Additionally, LPS can either induce or suppress host immune responses [58], with effects varying among different fish species [58,59,60] and primarily being dose-dependent. While increased dosage often correlates with stronger and longer-lasting responses, there are studies indicating otherwise [60]. Moreover, the source of LPS can influence host responses due to morphological differences in the molecule depending on the bacterial species [58]. Furthermore, some researchers have noted that salinity can suppress immune responses, thus mitigating LPS-induced toxicity [61]. The binding of divalent cations (Mg^2+^, Ca^2+^, Ba^2+^) to LPS alters its physicochemical properties and biological activity. These effects stem from the inability of the altered LPS structure to interact with binding proteins such as lipopolysaccharide-binding protein (LBP) and CD14 found in the host [62], which are crucial in LPS recognition by TLR4 receptors, ultimately leading to the expression and synthesis of immune effectors like antimicrobial peptides (AMPs) [48].

In aquaculture systems, fish are exposed to a wide variety of environmental stressors [63,64]. Management, exposure to aquatic microbiota, high stocking density, and alteration of physicochemical water parameters, such as pH, dissolved oxygen, and salinity, cause alterations in the quantity and composition of fish mucus [39,41,65]. Among water quality parameters, DO is the main limiting factor in the cultivation of aquaculture species. In Chilean meagre, the antibacterial effect of fish exposed to acute and intermittent hypoxia was reduced by 20–30% against *V. anguillarum* and to a lesser extent against *V. ordalii*. Additionally, a decrease in lysozyme activity levels from 60.00 ± 11.31 U mL^−1^ to 30.00 ± 2.83 U mL^−1^ was detected in the mucus of fish exposed to intermittent hypoxia events for 6 weeks. Oxygen deficiency can also result in mucosal barrier injury and increased permeability [33,64], favoring opportunistic pathogen infection and compromising animal health. However, some teleost fish species have developed tolerance mechanisms [66], being able to manage low oxygen availability. Similarly, the decrease in mucus antibacterial activity observed in *C. gilberti* under hypoxic conditions did not imply the loss of mucus defense capacity in larger juveniles, which exhibited an antibacterial potential of 42.3% against *V. anguillarum* and 24.8% against *V. ordalii*. Moreover, this was followed by a noticeable increase in mucus activity shortly after returning from hypoxic to normoxic conditions. Even the normal activity values increased by 33–37% in fish exposed to an acute hypoxia event and by 19.8% in fish exposed to chronic hypoxia. Water reoxygenation also enhanced lysozyme activity in unstimulated fish. After 24 h of returning to normoxia, 17 g juvenile fish previously exposed to acute hypoxia exhibited lysozyme activity of 32.00 ± 0.00 U mL^−1^, whereas the normoxia group showed levels of 4.00 ± 0.00 U mL^−1^. This effect was observed even 6 days later in 178 g juveniles exposed to chronic hypoxia, with lysozyme activity levels of 102.00 ± 36.77 U mL^−1^ compared to 54.00 ± 2.83 U mL^−1^ in the normoxia group. These responses during deoxygenation and reoxygenation suggest the existence of adjustment mechanisms to the environmental condition, as observed with other stressors in other species [34,67]. These reversible mechanisms could be regulated by the activation of a hypoxia-inducible factor (HIF-1α) [64,68,69,70], favoring hypoxia tolerance and maintaining tissue integrity and homeostasis, as well as the proper function of phagocytic cells. In this context, hypoxia did not alter the peroxidase content of *C. gilberti* mucus, despite reports of decreased activity of antioxidant enzymes and lysozyme in other species [33,71]. It has been documented that hypoxia can also impact the respiratory burst of macrophages, leading to the generation of large amounts of ROS [33]. Therefore, maintaining adequate levels of peroxidase activity could help minimize the excessive accumulation of ROS, thereby preventing oxidative damage to fish tissues.

The inoculation of inactivated *V. anguillarum* similarly induced peroxidase activity in both fish maintained under normoxia conditions and those subjected to an acute hypoxia event. However, the group exposed to hypoxia showed a notably more pronounced decrease in enzyme activity within 24 h. In contrast, LPS, administered by immersion or by intraperitoneal inoculation, only increased peroxidase levels in normoxic fish, suggesting that hypoxia affects the meagre response mechanisms to the endotoxin. Furthermore, LPS produced a reduction in the antibacterial potential of mucus by 20–21% against *V. anguillarum* and 11–16% against *V. ordalii*. Administering inactivated bacteria also failed to enhance the antibacterial response of mucus under conditions of low oxygen availability. Specifically, a notable decline in activity was observed, with a 49% reduction against *V. anguillarum* and a 36.2% decrease against *V. ordalii*. These findings underscore, akin to observations in other commercially significant fish species, the adverse impact of hypoxia events on vaccine effectiveness [72]. However, in the case of corvina, this effect proved transient. Fish subjected to acute hypoxia and subsequently bath-inoculated exhibited a return to baseline levels within 24 h, akin to unstimulated fish. Notably, neither lipopolysaccharide (LPS) nor bacterin induced activity in *C. gilberti* mucus. Additionally, lysozyme activity persisted in a pattern consistent with mucus response against *Vibrio* spp. This sustained consistency across the study suggests a pivotal role of lysozyme in the *C. gilberti* mucus response to pathogens, warranting its consideration as a potential biomarker for the *C. gilberti* immune response. In addition, these results advocate for exploring alternative strategies to immunological induction during periods of low oxygen availability in *C. gilberti* juvenile cultures. In this context, the levels of antibacterial activity in mucus observed under various conditions studied, coupled with *C. gilberti*’s capacity to regulate and restore these levels following hypoxia-induced stress periods, underline the importance of implementing measures aimed at preserving and enhancing mucosal integrity and epidermal mucus production to optimal and sufficient levels, thereby ensuring protection against potential pathogens.

This study is the first to analyze the defensive role of *C. gilberti* epidermal mucus under specific environmental conditions of fish cultivation. While further research is needed, our findings reveal the protective efficacy of *C. gilberti* mucus against pathogens, evident even under hypoxia-induced stress conditions. The general resilience demonstrated by *C. gilberti* towards fluctuations in dissolved oxygen (DO) concentration aligns with the species’ tolerance to other stressors documented in previous studies on *C. gilberti* [73]. In these studies, high stocking densities did not induce chronic stress in the animals nor did they impact their growth and physiological responses, even under acute hypoxic conditions. These observations underscore the species’ ability to tolerate diverse stressors studied thus far.

Subsequently, the identification and isolation of active components of mucus with antimicrobial function will enable the determination of immunological markers, providing non-invasive tools for monitoring fish health conditions and evaluating environmental factors for cultivation. In line with this objective, our research group has undertaken proteomic characterization of epidermal mucus from three native Chilean species as part of the Program for the Diversification of Chilean Aquaculture, which includes the Chilean meagre. These findings are presently undergoing review, and we anticipate that they will complement the results obtained in this study.

## 5. Conclusions

The findings presented in this study reveal an age-dependent antibacterial activity within the mucus of *C. gilberti* against *V. anguillarum*. Additionally, both lysozyme and peroxidase activity levels also increase with the age and/or size of juvenile fish. Notably, *C. gilberti* demonstrated exceptional resilience to fluctuations in dissolved oxygen concentration, enduring levels as low as 2 mg/L continuously for 3 h and intermittently over a period of 6 weeks. Despite hypoxia exerting an influence on the antibacterial response of the mucus, resulting in reduced effectiveness against *V. anguillarum* and *V. ordalii*, the mucus retained activity under both normoxic and hypoxic conditions. Remarkably, *C. gilberti* was able to swiftly restore mucus activity levels following hypoxic events. Conversely, hypoxia did not influence peroxidase activity, although chronic hypoxia impeded its induction by PAMPs. Similarly, hypoxia affected the immunological induction of *C. gilberti*, while immunostimulation of fish via intraperitoneal injection successfully enhanced the antibacterial effect of the mucus under normoxic conditions. Interestingly, the pattern of lysozyme activity remained consistent with antibacterial activity against *Vibrio* spp., highlighting its pivotal role in the antibacterial response of mucus. Additional studies will be required to evaluate whether chronic hypoxia without intermittent interruptions could result in more pronounced alterations in immune response.

## Figures and Tables

**Table 1:** Equivalence between bacterial concentration (CFU mL^−1^) and optical density at 600 nm for *V. anguillarum* 507 and *V. ordalii* DSM 19621. Data obtained in the laboratory.

Bacterial Strain	Optical Density at 600 nm	UFC mL^−1^
*Vibrio anguillarum* 507	0.380	1.00 × 10^9^
*Vibrio ordalii* DSM 19621	0.100	1.00 × 10^8^

**Table 2:** Lysozyme activity (U mL^−1^) in Chilean meagre epidermal mucus and equivalent amount of chicken egg lysozyme (µg) per 100 µg of mucus total proteins. Results obtained from samples collected during acute and chronic hypoxia experiments.

Experiment	Time Point	Treatment	Lysozyme Activity (U mL^−1^)	Equivalent Amount of Egg Lysozyme (µg per 100 µg of Sample)
Acute hypoxia	After hypoxia (3 h)	Normoxia	ND	-
		Hypoxia	4.00 ± 5.66	0.09 ± 0.13
	After immunostimulation	N-PBS	10.00 ± 2.83	0.19 ± 0.01
		N-VA	ND	-
		H-PBS	ND	-
		H-VA	ND	-
	24 h post- immunostimulation	N-PBS	4.00 ± 0.00 ^a^	0.16 ± 0.00
		N-LPS	2.00 ± 2.83 ^a^	0.08 ± 0.12
		N-VA	ND ^a^	-
		H-PBS	32.00 ± 0.00 ^b^	0.29 ± 0.00
		H-LPS	ND ^a^	-
		H-VA	40.00 ± 5.66 ^b^	0.33 ± 0.03
Chronic hypoxia	After intermittent hypoxia (6 weeks)	Normoxia	60.00 ± 11.31	0.42 ± 0.05
		Hypoxia	30.00 ± 2.83	0.28 ± 0.01
	6 days post- immunostimulation	N-PBS	54.00 ± 2.83	0.39 ± 0.01
		N-LPS	146.00 ± 8.49	0.81 ± 0.04
		H-PBS	102.00 ± 36.77	0.61 ± 0.17
		H-LPS	70.00 ± 8.49	0.47 ± 0.04

Results expressed as mean ± SD. The letters indicate statistically significant differences between groups (*p* ≤ 0.05) at the same sampling time. N: normoxia; H: hypoxia; PBS: non-stimulated group; LPS: group inoculated with LPS; VA: group inoculated with the inactivated vaccine. ND: not detected, values ≤ 0.

**Table 3:** Peroxidase content of the Chilean meagre mucus samples from acute and chronic hypoxia experiments.

Experiment	Time Point	Treatment	Peroxidase Activity (U mg^−1^)	Amount of Peroxidase per 100 µg of Total Protein Extracts (µg)
Acute hypoxia	After hypoxia (3 h)	Normoxia	0.73 ± 0.42	0.07 ± 0.03
		Hypoxia	0.97 ± 0.26	0.09 ± 0.02
	After immunostimulation	N-PBS	0.20 ± 0.06 ^a^	0.04 ± 0.00
		N-VA	0.46 ± 0.01 ^bc^	0.05 ± 0.00
		H-PBS	0.33 ± 0.01 ^ac^	0.05 ± 0.00
		H-VA	0.60 ± 0.01 ^b^	0.06 ± 0.00
	24 h post- immunostimulation	N-PBS	0.30 ± 0.05 ^ab^	0.04 ± 0.00
		N-LPS	0.85 ± 0.03 ^c^	0.08 ± 0.00
		N-VA	0.29 ± 0.06 ^ab^	0.04 ± 0.00
		H-PBS	0.34 ± 0.02 ^a^	0.05 ± 0.00
		H-LPS	0.14 ± 0.01 ^bd^	0.03 ± 0.00
		H-VA	0.10 ± 0.01 ^d^	0.03 ± 0.00
Chronic hypoxia	After intermittent hypoxia (6 weeks)	Normoxia	3.26 ± 0.00	0.52 ± 0.00
		Hypoxia	3.32 ± 0.03	0.53 ± 0.00
	6 days post- immunostimulation	N-PBS	0.05 ± 0.01 ^a^	0.03 ± 0.00
		N-LPS	2.57 ± 0.01 ^b^	0.42 ± 0.00
		H-PBS	0.65 ± 0.04 ^c^	0.12 ± 0.01
		H-LPS	0.74 ± 0.06 ^c^	0.13 ± 0.01

Results expressed as mean ± SD. The letters indicate statistically significant differences between groups (*p* ≤ 0.05). N: normoxia; H: hypoxia; PBS: non-stimulated group; LPS: group inoculated with LPS; VA: group inoculated with the inactivated vaccine.

## Data Availability

Dataset available on request from the authors.
